# Aptamer Turn-On SERS/RRS/Fluorescence Tri-mode Platform for Ultra-trace Urea Determination Using Fe/N-Doped Carbon Dots

**DOI:** 10.3389/fchem.2021.613083

**Published:** 2021-03-15

**Authors:** Chongning Li, Jiao Li, Aihui Liang, Guiqing Wen, Zhiliang Jiang

**Affiliations:** ^1^ State Key Laboratory for Chemistry and Molecular Engineering of Medicinal Resources, School of Chemistry and Pharmaceutical Science, Guangxi Normal University, Guilin, China; ^2^ Key Laboratory of Ecology of Rare and Endangered Species and Environmental Protection (Guangxi Normal University), Ministry of Education, Guilin, China; ^3^ Guangxi Key Laboratory of Environmental Pollution Control Theory and Technology for Science and Education Combined with Science and Technology Innovation Base, Guilin, China

**Keywords:** Fe/N-doped carbon dots, catalysis amplification, aptamer, surface-enhanced Raman scattering, resonance Rayleigh scattering, fluorescence

## Abstract

Sensitive and selective methods for the determination of urea in samples such as dairy products are important for quality control and health applications. Using ammonium ferric citrate as a precursor, Fe/N-codoped carbon dots (CD_FeN_) were prepared by a hydrothermal procedure and characterized in detail. CD_FeN_ strongly catalyzes the oxidation of 3,3′,5,5′-tetramethylbenzidine (TMB) by H_2_O_2_ to turn on an indicator molecular reaction, forming an oxidized tetramethylbenzidine (TMB_ox_) probe with surface-enhanced Raman scattering, resonance Rayleigh scattering, and fluorescence (SERS, RRS, and FL) signals at 1,598 cm^−1^, 370 nm, and 405 nm, respectively. The urea aptamer (Apt) can turn off the indicator reaction to reduce the tri-signals, and the addition of urea turns on the indicator reaction to linearly enhance the SERS/RRS/FL intensity. Thus, a novel Apt turn-on tri-mode method was developed for the assay determination of ultra-trace urea with high sensitivity, good selectivity, and accuracy. Trace adenosine triphosphate and estradiol can also be determined by the Apt-CD_FeN_ catalytic analytical platform.

## Introduction

Urea is a naturally occurring metabolite of nitrogen-containing compounds (Dervisevic et al., 2018). It has many applications and can be found in both fertilizer and dermatological cream. Because it can in some cases cause adverse effects, urea detection methods are common in clinical chemistry, agriculture, and biology. Currently, urea detection methods include surface-enhanced Raman scattering (SERS), colorimetric, electrochemical, and fluorescence (FL) approaches, (Safitri et al., 2017; Migliorini et al., 2018), most of which operate in a single mode with low sensitivity. The development of urea detection methods with enhanced sensitivity, such as highly sensitive SERS, FL, and resonance Rayleigh scattering (RRS) tri-mode reactions, has therefore attracted significant attention.

CDs are carbon-based nanomaterials with good water solubility and surfaces that can be easily functionalized with various organic polymers, inorganic moieties, or biological species (Karthikeyan et al., 2019). They have been widely used in chemistry, environmental science, food science, and biotechnology (Rong et al., 2018; Zheng et al., 2018). CDs can be doped with inorganic metal ions, which is an effective method for improving their optical and electrical properties. For example, Fe is an abundant element that is compatible with carbon-based materials. Fe-doped CDs (CD_Fe_) are suitable for various prospective applications and can be made to fluoresce. Fluorescent CD_Fe_ have been prepared using a one-step hydrothermal carbonization method, with methylthymol blue sodium salt and FeCl_3_∙6H_2_O as precursors (Zhu et al., 2019). These CD_Fe_ were employed in a glucose/CD_Fe_ ratio FL sensing system to quantify H_2_O_2_ and glucose presence in the concentration ranges of 0–133 and 0–300 μM with limits of detection (LODs) of 0.47 and 2.5 μM, respectively. N and Fe-containing CDs (CD_FeN_) have been used to measure dopamine content by colorimetry and fluorometry (Wang et al., 2016). Furthermore, L-tartaric acid, urea, and FeCl_3_∙6H_2_O have been used as precursors in a solvothermal procedure to synthesize CD_FeN_ for the immunosorbent spectrophotometric detection of carcinoembryonic antigens at levels as low as 0.1 pg/ml (Yang et al., 2017). Ethylenediamine tetraacetate and iron nitrate have been used as carbon and iron sources, respectively, to obtain CD_Fe_ through a one-step hydrothermal carbonization, which provided a favorable electron acceptor near the CD_Fe_ and produced high quenching efficiency (Zhuo et al., 2019). This FL response can quantify dopamine in the range of 0.01–50 μM with an LOD of 5 nM. However, to the best of our knowledge, there have been no reports on the use of a single precursor to prepare CD_FeN_ or the catalytic amplification of tri-signals and their utilization to detect trace urea using an aptamer (Apt).

Apts are ideal for detecting target molecules, such as urea, as they bind to a chosen molecule. Moreover, they are easy to synthesize and modify, chemically stable, and can be stored for long periods. They have already been used to specifically capture metal ions and small organic molecules in recent trace substance analyses (Zhou and Rossi, 2017). For example, the use of a single-labeled multifunctional probe comprising a Cd(II)-specific Apt for measuring Cd(II) by FL with an LOD of 2.15 nM has been demonstrated (Zhu et al., 2017). A label-free fluorescent Apt sensor for tetracycline junction Apts and thiazole orange for the selective and sensitive FL detection of 0.05–100 μg/ml tetracycline has also been established (Sun et al., 2018). Furthermore, a label-free and off-FL method for the quantitative detection of 0.7–10 nmol/L kanamycin based on functional molecular beacons was recently developed (Zhu et al., 2018). The interaction between silver nanoparticles (AgNPs) and CdTe quantum dots to detect 0.1–30 nM adenosine has also been successfully demonstrated (Song et al., 2018). In conjunction with SERS, Apts can contribute significantly to the detection of trace urea.

SERS is a molecular spectroscopy technique based on Raman scattering and local surface plasmon resonance of nanoparticles. It has been used in numerous fields, including nanomaterial research, bioanalysis, and food testing (Chen et al., 2017b; Deng et al., 2017; Li et al., 2018b). Graphene oxide nanoribbons with a strong catalytic effect on the reduction of HAuCl_4_ by H_2_O_2_, forming gold nanoparticles with SERS activity, were developed (Li et al., 2018a). Coupling with an Apt reaction allowed for the quantitative analysis of 2–75 nmol/L Pb(II) with the molecular probe Victoria Blue B. The thickness of Fe_2_O_3_ coatings on Fe_2_O_3_ at graphene nanostructures was adjusted by changing the number of Fe_2_O_3_ atomic layer deposition cycles (Zhang et al., 2017). Fe_2_O_3_ was deposited on a graphene surface, and combination with an Apt yielded a simple, fast, and sensitive electrochemical Apt sensor for the detection of 1.0 × 10^−11^–4.0 × 10^−9^ M thrombin with an LOD of 1.0 × 10^−12^ M.

Dual-mode molecular probes (e.g., FL/colorimetry (Li et al., 2017), FL/light scattering (Liu et al., 2017), and FL/SERS (You et al., 2017)) also play a pivotal role in this experiment. They have attracted widespread attention owing to their simplicity while displaying higher sensitivity than traditional optical sensors. A SERS/FL dual-mode nanosensor with a signal transduction mechanism based on the conformational transformation of human telomeric G-quadruplex was developed (Liu et al., 2015). The nanosensor exhibited an excellent SERS/FL response to the complementary strand of the G-quadruplex. Based on T-Hg^2+^-T coordination chemistry, the sensor can be used to detect Hg^2+^ at an LOD as low as 1 ppt. Zou et al. (Zou et al., 2015) used graphene quantum dot tags to design a new dual-mode immunoassay method based on SERS and FL to detect tuberculosis through a newly developed linear comparison sensing platform for the antigen CFP-10. The sandwich-type immunoassay uses a dual-mode nanoprobe to recognize SERS signals and FL images in a highly sensitive and selective manner with an LOD of 0.0511 pg/ml. However, there have been few reports on tri-mode methods with the nanocatalytic amplification of signals. For example, Li et al. (Li et al., 2018b) reported the colorimetric/FL/SERS tri-mode sensing of nitrite based on a Griess-reaction-modulated gold nanorod–Azo–nanogold assembly. Colorimetric and FL detection were carried out in solution, whereas SERS was performed on a solid substrate, achieving LODs of 0.05, 0.01, and 0.0008 μM by their respective methods.

The gold nanoparticle-TMB-H_2_O_2_ (TMB = 3,3′,5,5′-tetramethylbenzidine) system can be used as an ultrasensitive colorimetric pH indicator, with the gold nanoparticles acting as a catalyst to mimic the function of horseradish peroxidase (Deng et al., 2016). In this catalytic reaction, the absorbance of the yellow product at 450 nm remained linear in the pH range of 6.40–6.60, and the LOD of urea was 5 μM. A nanoparticle-based urea FL sensing scheme has also been reported (Shao et al., 2015). Graphene quantum dots displayed pH-sensitive green FL upon photoexcitation at 460 nm, and urease-catalyzed urea hydrolysis led to a local increase in pH and gradual FL quenching. This approach can be used to quantify urea in the concentration range of 0.1–100 mM with an LOD of 0.01 mM SnO_2_ quantum dot/reduced graphene oxide composites were used to prepare enzyme-free ultrasensitive urea sensors (Dutta et al., 2014). These SnO_2_ quantum dots can be modified on the reduced graphene oxide layer, and urea can be detected by evaluating the sensor characteristics. The electrode prepared with the composite was sensitive to urea in a concentration range of 1.6 × 10^−14^–3.9 × 10^−12^ M with an LOD of 11.7 mM. SERS technology is a highly sensitive detection technology, and its signal enhancement mainly depends on the probe molecule and substrates. The structure and small scattering cross-section of urea molecules cannot directly produce SERS signals on gold or silver substrates. Therefore, urea molecules are ineffective as probe molecules. In this study, we developed an indirect method to detect ultra-trace urea. We found that CD_FeN_ catalyzes the oxidation of TMB to form TMB_ox_, an effective SERS probe. Furthermore, urea Apt can inhibit the catalysis of CD_FeN_ and simultaneously reduce its FL. The prepared CD_FeN_ was used to catalyze the formation of TMB_ox_ from H_2_O_2_-TMB, from which a novel, highly sensitive, and selective Apt reaction turn-on SERS/RRS/FL tri-mode analytical platform was developed for the detection of ultra-trace small organic molecules, as demonstrated herein with urea.

## Materials and Methods

### Instruments and Reagents

#### Instruments

A Hitachi F-7000 FL spectrophotometer (Hitachi High-tech), TU-1901 dual-beam ultraviolet-visible spectrophotometer (Beijing General Analysis General Instruments), and DXR smart Raman spectrometer (Thermo, United States) with an excitation wavelength of 633 nm, laser power of 3.5 mW, slit width of 50 μm, and acquisition time of 5 s were used to measure the CD system signals. The following were used to synthesize and characterize the CD systems: a desktop centrifuge (Zhuhai Heima Medical Instrument); ultrasonic cleaner (Shanghai Kedao Ultrasonic Instrument); SYZ-550 quartz sub-boiling distilled water device (Jiangsu Crystal Glass Instrument Factory); 79-1 magnetic heating stirrer (Jiangsu Zhongda Instrument Factory); HH-S2 electric heating thermostatic water bath (Jintan Dadi Automation Instrument Factory); KP-216 air energy light wave furnace (Zhongshan Qiaokang Electric Manufacturing, rated power 1200 W); pH meter (Mettler-Toledo Instruments Shanghai); Nano-2s nanometer particle size and zeta potential analyzer (Malvern, United Kingdom); and an S-4800 field emission scanning electron microscope (SEM; Hitachi Hi-tech).

#### Reagents

Urea ssDNA Apt (Apt_urea_) with the sequence 5′-3′ CAC AAG CAC AGA CAG CTG TTC CAC AT was acquired from Shanghai Biotech Biological, Shanghai, China. Ammonium ferric citrate (Sinopharm Group Chemical Reagent, Shanghai, China), 30% H_2_O_2_ (10^5^ times dilution, Shanghai Chemical Reagent, Shanghai, China), 0.1 mol/L HCl, 0.1 mol/L Tris solution, 5.05 mmol/L pH 4.4 Tris-HCl (concentration based on the amount of HCl: 500 μL of 0.1 mol/L Tris and 505 μL of 0.1 mol/L HCl were prepared in 10 ml of ultra-pure water), and 0.5 mmol/L TMB (storage: 2–8°C, T818493-5 g, CAS: 54827-17-7, Shanghai McLean Biochemical Technology, Shanghai, China) were employed for synthesis and analyses. TMB (0.012 g) was weighed and dissolved in 100 ml of an ethanol solution (ethanol: water = 1:1) to obtain the stock solution. Water (44 ml) was added to an Erlenmeyer flask and combined with 2 ml of 10 mmol/L AgNO_3_, 2.0 ml of 100 mm/L trisodium citrate, 600 μL of 30% H_2_O_2_, and 600 μL of 0.1 mol/L NaBH_4_ added sequentially under stirring until the color turned blue. AgNPs were added to the analysis system as an SERS enhancement substrate. Without the addition of AgNPs, the system could generate very weak Raman signals. The prepared blue AgNP gel was then immediately transferred into a light-wave oven and heated at 250°C for 10 min to obtain an orange-red transparent AgNP gel. After cooling naturally, water was added to the product to obtain a total volume of 50 ml at a concentration of 4.0 × 10^−4^ mol/L AgNPs. All reagents were analytical grade, and all experiments employed secondary distilled water.

#### Preparation of CD_Fe_ and CD_FeN_

Ferric citrate or ammonium ferric citrate powder (0.02 g) was accurately weighed and dissolved in 30 ml of ultra-pure water. The dark yellow solution was then transferred to a polytetrafluoroethylene-based autoclave. After sealing, a hydrothermal reaction was performed in a muffle furnace at the optimal temperature of 180°C for 3 h. After the reaction was complete, ice water was used to cool the product to room temperature to obtain a brown solution. The brown-yellow solution was centrifuged at 10000 rpm for 10 min to remove the precipitate, and the supernatant was dialyzed against a dialysis bag with a molecular weight cutoff of 3,500 Da for 12 h to obtain 0.67 mg/ml CD_Fe_ or CD_FeN_, which was diluted for further use.

### Optimization and Characterization of CDs

CD synthesis was optimized based on the product FL intensity. As shown in Supplementary Figure S1, FL was the strongest when the reaction was performed at 180°C for 30 min. The amount of precursor was selected according to the strength of the catalytic effect of the CDs. Generally, the amount corresponding to the maximum CD concentration and the maximum slope of the FL intensity curve of the TMB oxidation product were selected. The experimental results indicated that 0.02 g of ammonium ferric citrate yielded CDs with the best catalytic effect. Compared with reported procedures for the preparation of CD_Fe_ (Supplementary Table S1) (Faraji et al., 2018; Yue et al., 2019; Zhang et al., 2019; Yadav et al., 2020), our procedure is simpler and requires a shorter hydrothermal reaction time. In addition, only one reagent, ammonium ferric citrate, was used to prepare CD_FeN_.

Five molecular techniques were used to characterize the CDs. The FL intensity of different CD_Fe_ concentrations in a Tris-HCl buffer solution was measured. Under the conditions of voltage = 500 V, excitation slit = emission slit = 10 nm, and λ_ex_ = 305 nm, the system generated a FL peak at 420 nm. With increasing CD_Fe_ concentration, the FL intensity gradually increased (Supplementary Figure S2A). The FL intensity of the CD_FeN_-Tris-HCl system at different concentrations was measured. The system generated an FL peak at 420 nm, which increased gradually in intensity with increasing CD_FeN_ concentration (Supplementary Figure S2B). The CD_FeN_ FL was stronger than that of CD_Fe_ due to the doped N element. The RRS intensity of the CD_Fe_/CD_FeN_-Tris-HCl system with different concentrations was measured. Under the conditions of voltage = 350 V and excitation slit = emission slit = 5 nm, the system produced a strong RRS peak at 375 nm, the intensity of which gradually increased with CD_Fe_/CD_FeN_ concentration (Supplementary Figures S2C,D). The CD_FeN_ RRS was stronger than that of CD_Fe_ due to the resonance between the large π-bond electrons and doped Fe electrons of the CDs. The UV-vis absorbance intensity of the CD_FeN_/CD_Fe_-Tris-HCl system was measured. As the concentration of CD_FeN_/CD_Fe_ increased, an absorption peak appeared at 320 nm (Supplementary Figures S2E,F). The absorbance of both CDs was similar due to the low sensitivity of the spectrophotometric method.

A 0.025 g/ml CD_Fe_ solution (10 ml) was placed in a material tray, pre-frozen in a vacuum drying freezer cold trap for 5 h, and dried at 0.1 Pa for 24 h. The obtained solid sample and the precursor materials (ferrocene powder A, ferric ammonium citrate powder, and potassium bromide) were mixed in equal amounts and ground uniformly in an agate mortar to prepare powder tablets for infrared spectral analysis. The infrared spectra of ammonium ferric citrate (Supplementary Figures S2G) contained strong peaks at 3,189 cm^−1^ (O-H stretching); 1,616 cm^−1^ (C=C conjugate stretching); 1,394 cm^−1^ (CO_2_^−^ symmetric stretching); 1,250 and 1,066 cm^−1^ (C-O stretching); and 909, 851, and 642 cm^−1^ (C=C-H bending). The infrared spectra of CD_FeN_ (Supplementary Figure S2H) contained strong peaks at 3,411 cm^−1^ (O-H telescopic vibration); 1,617 cm^−1^ (C=C conjugate telescopic vibration), 1,384 cm^−1^ (CO_2_^−^ symmetric telescopic vibration), 1,049 cm^−1^ (C-O telescopic vibration), 560 cm^−1^ (C-H out-of-plane bending), and 470 cm^−1^ (C=C-H bending). The infrared spectra of the CDs and the corresponding precursors were significantly different. The infrared peak at 3,411 cm^−1^ (O-H stretching vibration) indicated the presence of hydroxyl groups in the CDs, confirming that the precursor material was successfully modified and had good water dispersion. Raman spectra of both the CD_FeN_ and CD_Fe_ solutions were examined, and no Raman peaks were observed. Using AgNPs in solution as a SERS substrate, weak SERS peaks were recorded (as shown in Supplementary Figures 2SI,J) at 1,160 and 1,620 cm^−1^ for CD_FeN_ and 1,620 cm^−1^ for CD_Fe_.

### Experimental Procedures

In a 5 ml stoppered graduated test tube, 200 μL of a 0.67 μg/ml CD_Fe/N_ solution, 100 μL of a 0.1 mmol/L H_2_O_2_ solution, 100 μL of a 0.5 mmol/L TMB solution, 250 μL of a 5.05 mmol/L pH 4.07 Tris-HCl solution, 200 μL of a 0.1 μmol/L Apt solution, and an appropriate amount of a urea solution were added sequentially. Water was then added to obtain a final volume of 1.5 ml. The reaction was performed at 50°C in a water bath for 30 min and then terminated by placing it in an ice water bath. Subsequently, 400 μL of a 0.4 mmol/L AgNP solution and then water was added to reach a total volume of 2 ml. Finally, the SERS and FL/RRS spectra were acquired using a Raman spectrometer and FL spectrophotometer, respectively.

## Results and Discussion

### Methodology

**FIGURE 1:**
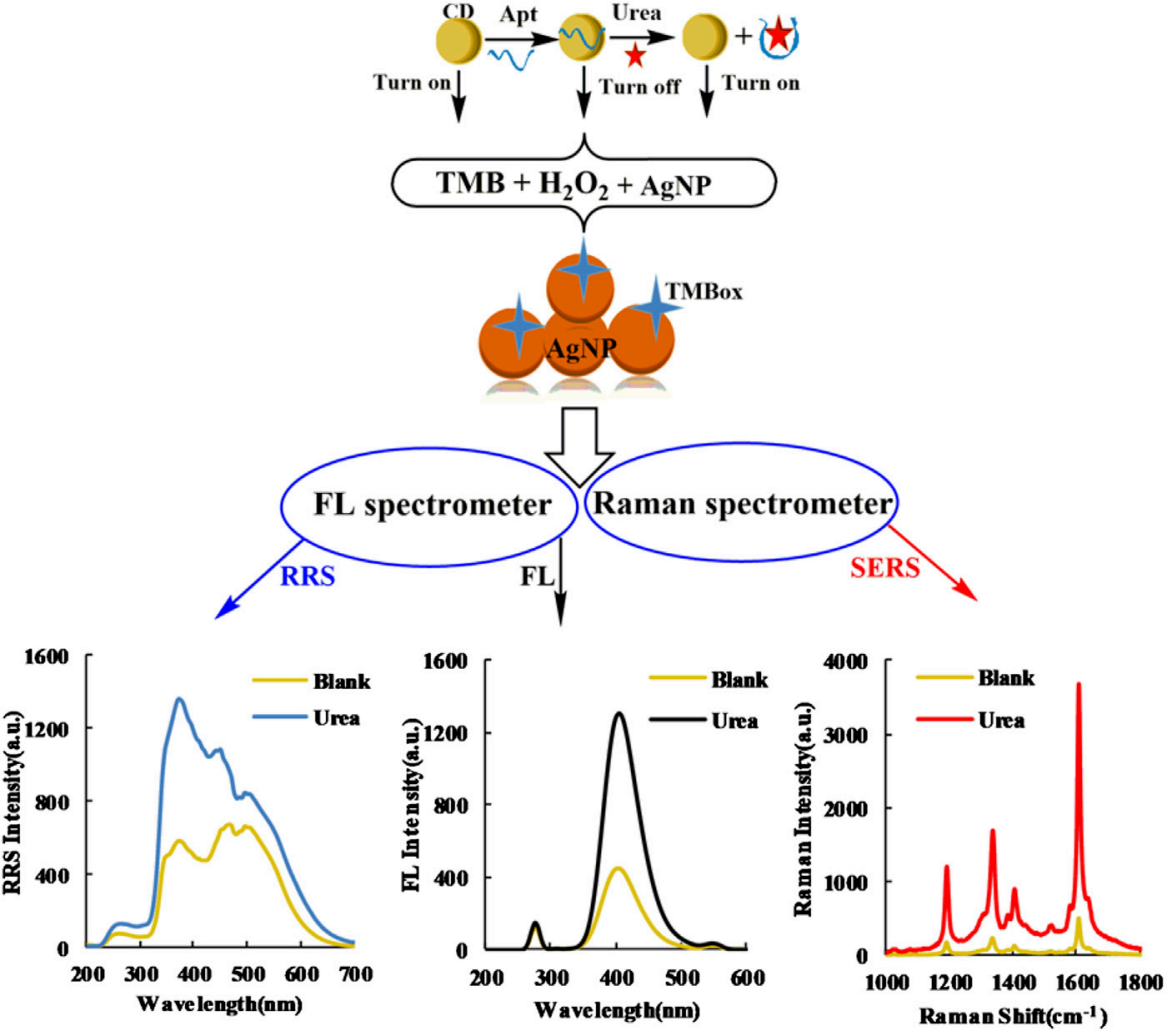
Schematic of SERS/RRS/FL tri-mode method to detect ultra-trace urea coupling Apt_urea_ with CD_FeN_ catalysis.

In the Tris-HCl buffer solution, the H_2_O_2_-TMB reaction was slow because the transfer of redox electrons is difficult between H_2_O_2_ and TMB. CD_FeN_ is rich in π-electrons and Fe metal electrons, which can enhance the redox electron transfer to effectively catalyze the formation of large π-bond TMB_ox_ in the H_2_O_2_-TMB system and generate a strong FL signal to turn on the FL indicator reaction. Apt_urea_ and the CDs form a complex morphology that suppresses the catalytic performance of the CDs, which thus reduces the system FL signal to turn off the indicator reaction. Based on the three-dimensional structure of Apt_urea_ and its flexibility, the spatial structure formed by the extension of the nucleic acid chain in solution affords a large contact area with urea, causing the CDs to change to a free state. This restores the catalytic activity to turn on the reaction, leading to an increase in the FL signal of the system. Within 3.33–20 nmol/L, the change in FL intensity displayed a linear relationship with urea concentration and TMB_ox_ exhibited good SERS activity on the AgNP substrates; the SERS signal of the system linearly increased within this concentration range. Thus, two FL and SERS analysis methods for urea were developed based on this CD_FeN_ catalytic amplification reaction. Because the formed TMB_ox_ results in AgNP aggregation, the RRS intensity also increased linearly with urea concentration. As a result, a simple, sensitive, Apt-mediated CD_FeN_ catalytic reaction with SERS, FL, and RRS tri-mode signals was established for the determination of urea (Figure 1).

### SERS Analysis of CD-H_2_O_2_-TMB-Apt-Urea Nanocatalytic Systems

**FIGURE 2:**
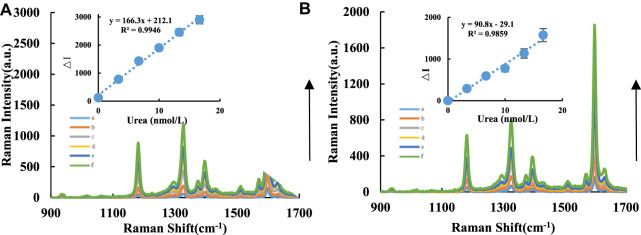
SERS spectra of CD-H_2_O_2_-TMB-Apt-AgNP analysis system. A/B compared the catalytic effects between CD_Fe_ and CD_FeN_ in the urea analysis system. **(A)**: CD_Fe_-H_2_O_2_-TMB-Tris-HCl-Apt-Urea-AgNPs system, a: 0.04 μg/ml CD_Fe_+5.0 μmol/L H_2_O_2_+ 0.03 mmol/L TMB+0.51 mmol/L PH = 4.07 Tris-HCl+10 nmol/L Apt+ Urea+0.08 mmol/L AgNPs; b: a+3.33 nmol/L Urea; c: a+6.66 nmol/L Urea; d: a+9.99 nmol/L Urea; e: a+13.32 nmol/L Urea; f: a+16.65 nmol/L Urea. **(B)**: CD_FeN_-H_2_O_2_-TMB-Tris-HCl-Apt-Urea-AgNPs system, a: 0.04 μg/ml CD_FeN_+ 5.0 μmol/L H_2_O_2_+ 0.03 mmol/L TMB+ 0.51 mmol/L PH = 4.07 Tris-HCl+10 nmol/L Apt+ Urea+0.08 mmol/L AgNPs; b: a+3.33 nmol/L Urea; c: a+6.66 nmol/L Urea; d: a+9.99 nmol/L Urea; e: a+13.32 nmol/L Urea; f: a+16.65 nmol/L Urea.

The SERS signal mainly arose from the TMB_ox_ molecule when AgNPs were added to the analysis system as a substrate. AgNPs were added as a uniformly dispersed colloidal system, and probe molecules could be homogeneously adsorbed on the surface of bare AgNPs to produce SERS. No SERS signal was observed when only AgNPs were employed. Although changes in the chemical environment may cause a shift in the position of the SERS peak (e.g., the presence of urea), the observed SERS peak in this study is that of TMB_ox_. In a pH 4.07 Tris-HCl buffer solution at 50°C in a water bath, CD catalyzed the oxidation of TMB by H_2_O_2_, and the TMB_ox_ exhibited SERS activity. The addition of Apt_urea_ can wrap around the CDs, which inhibits their catalytic ability and reduces the formation of TMB_ox_ and the corresponding SERS intensity. When the target molecule urea was added, it specifically bound to the corresponding Apt_urea_ and released the CDs, restoring their catalytic activity. The Raman spectrum was obtained with a light source power of 2.5 mW and slit of 25.0 μm. The SERS intensity at 1,598 cm^−1^, $I_{1598{\text{cm}}^{-1}}$, was measured, the blank value ${\left(I_{1598{\text{cm}}^{-1}}\right)}_{0}$ without the urea solution was recorded, and $\Delta I_{1598{\text{cm}}^{-1}}=I_{1598{\text{cm}}^{-1}}-{\left(I_{1598{\text{cm}}^{-1}}\right)}_{0}$ was calculated. When AgNPs were added, stronger Raman peaks at 1,284, 1,356, and 1,598 cm^−1^ appeared. The CD systems showed strong Raman peaks at 1,183, 1,328, and 1,598 cm^−1^, and the SERS signal of the system increased linearly (Figures 2A,B).

### FL Spectra of Nanocatalytic and Apt System

**FIGURE 3:**
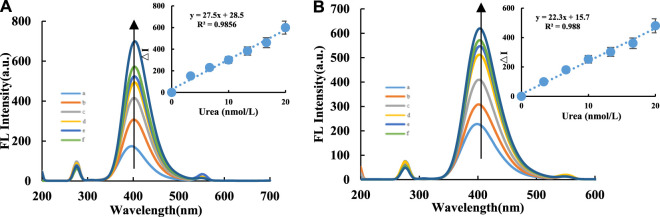
FL spectra of CD-H_2_O_2_-TMB analysis system. Without H_2_O_2_-TMB probe, A/B compared the fluorescence between CD_Fe_ and CD_FeN_ in the urea analysis system. **(A)**: CD_Fe_-H_2_O_2_-TMB-Tris-HCl-Apt-Urea system. a: 0.04 μg/ml CD_Fe_+ 6.7 μmol/L H_2_O_2_+ 0.03 mmol/L TMB+ 0.84 mmol/L PH = 4.4 Tris-HCl+16.65 nmol/L Apt; b: a+3.33 nmol/L Urea; c: a+6.66 nmol/L Urea; d: a+9.99 nmol/L Urea; e: a+13.32 nmol/L Urea; f: a+16.65 nmol/L Urea; g: a+20 nmol/L Urea; **(B)**: CD_FeN_-H_2_O_2_-TMB-Tris-HCl-Apt-Urea system, a: 0.04 μg/ml CD_FeN_+6.7 μmol/L H_2_O_2_+0.03 mmol/L TMB+ 0.84 mmol/L PH = 4.4 Tris-HCl+16.65 nmol/L Apt; b: a+3.33 nmol/L Urea; c: a+6.66 nmol/L Urea; d: a+9.99 nmol/L Urea; e: a+13.32 nmol/L Urea; f: a+16.65 nmol/L Urea; g: a+20 nmol/L Urea.

CDs are fluorescent nanomaterials, and in this study, the FL peak height at 405 nm was monitored instead of the peak area to approximate the FL intensity. This method is not very precise, but the calculations are easy; this caused the problem related to the change in the slope of adjacent points at both ends of the linear range. For the CD-H_2_O_2_-TMB nanocatalytic system, within a 0.02 – 0.14 μg/ml CD concentration range, the FL intensity increased from CDs more strongly catalyzing the oxidation of TMB by H_2_O_2_ (Supplementary Figures S3A,B). For the Apt inhibition system CD-HCl-H_2_O_2_-TMB-Apt, the CDs are wrapped when Apt_urea_ is added, which inhibits the ability of the CDs to catalyze H_2_O_2_-TMB, reduces TMB_ox_ formation, and thus decreases the FL intensity. FL spectra of the CD-H_2_O_2_-TMB-Apt system were measured with voltage = 350 V and excitation slit = emission slit = 10 nm. With increasing Apt_urea_ concentration, the FL intensity of the system gradually and linearly weakened (Supplementary Figures S3C,D). For the FL intensity of the CD_Fe_-Apt system, the conditions of voltage = 500 V, excitation slit = emission slit = 10 nm, and λ_ex_ = 305 nm generated a FL peak at 395 nm. With increasing Apt_urea_ concentration, the FL intensity of the system gradually decreased. For the CD_FeN_-Apt system, the conditions of voltage = 500 V, excitation slit = emission slit = 10 nm, and λ_ex_ = 310 nm generated a FL peak at 405 nm. With increasing Apt_urea_ concentration, the FL intensity of the system gradually decreased (Supplementary Figures S3E,F). Based on these results, Apt_urea_ can effectively wrap the CDs to linearly decrease the FL intensity of the system with increasing Apt_urea_ concentration, demonstrating the interaction between the CDs and Apt_urea_, and the applicability of the CDs as FL probes to identify the interaction. For the CD-H_2_O_2_-TMB-Apt-Urea system, when the target molecule urea was added, urea and Apt_urea_ formed a stable conjugate and released the CDs, thus restoring their catalytic activity. As a result, the FL intensity of the system gradually increased. Under the conditions of voltage = 350 V, excitation slit = emission slit = 10 nm, and λ_ex_ = 275 nm, the system generated a FL peak at 405 nm. Within 3.33 – 20 nmol/L, the FL intensity displayed a linear relationship with the urea concentration (Figures 3A,B).

### RRS Analysis of Nanocatalyst and Apt System

**FIGURE 4:**
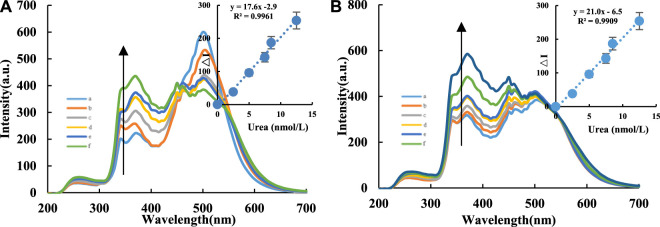
RRS spectra of CD-Tris-HCl-Apt analysis system. A/B compared the RRS analysis system of urea using CD_Fe_ and CD_FeN_, respectively. **(A)**: CD_Fe_-H_2_O_2_-TMB-Tris-HCl-Apt-Urea-AgNPs system, a: 0.04 μg/ml CD_Fe_ + 6.7 μmol/L H_2_O_2_+ 0.03 mmol/L TMB+ 0.84 mmol/L PH = 4.4 Tris-HCl+16.65 nmol/L Apt+0.08 mmol/L AgNPs; b: a+2.5 nmol/L Urea; c: a+5 nmol/L Urea; d: a+7.5 nmol/L Urea; e: a+8.5 nmol/L Urea; f: a+12.5 nmol/L Urea. **(B)**: CD_FeN_-H_2_O_2_-TMB-Tris-HCl-Apt-Urea-AgNPs system, a:0.04 μg/ml CD_FeN_ + 6.7 μmol/L H_2_O_2_+ 0.03 mmol/L TMB+ 0.84 mmol/L PH = 4.4 Tris-HCl+16.65 nmol/L Apt+0.08 mmol/L AgNPs; b: a+2.5 nmol/L Urea; c: a+5 nmol/L Urea; d: a+6 nmol/L Urea; e: a+7.5 nmol/L Urea; f: a+10 nmol/L Urea; g: a+12.5 nmol/L Urea.

RRS is a synchronous FL scanning technology where the excitation light wavelength is equal to the emission light wavelength (Δλ = λ_em_−λ_ex_ = 0). The signal intensity mainly originates from the scattering of excitation light by nanoparticles. The degree of aggregation of particles in the system and changes in particle size cause signal changes. For the CD_Fe_/CD_FeN_-Apt system, under the conditions of voltage = 400 V and excitation slit = emission slit = 5 nm, RRS peaks appeared at 370, 380 and 385 nm. The RRS intensity of the system gradually decreased with increasing Apt_urea_ concentration (Supplementary Figures S4A,B). Therefore, Apt_urea_ can effectively wrap the CDs to linearly decrease the RRS intensity of the system with increasing Apt concentration. In the absence of AgNPs, the CD-H_2_O_2_-TMB catalytic reaction of the system occurs, but the nanocatalyst CD concentration was low and the TMB concentration was very low (0.03 mmol/L). Thus, the TMB_ox_ molecules produced were low with a very weak RRS signal, resulting in weak overall RRS spectral intensities of the system. However, the CD-H_2_O_2_-TMB-AgNP system generated a strong RRS peak at 380 nm since the produced TMB_ox_ resulted in AgNP aggregation (Yao et al., 2019). As the CD concentration increased, the RRS intensity of the system gradually increased (Supplementary Figures S4C,D). For the CD-H_2_O_2_-TMB-Apt-Urea system, when the target molecule urea was added, urea and Apt_urea_ formed a stable conjugate and released the CDs. This restored the CD catalytic activity, causing the RRS intensity of the system to gradually increase. Under the conditions of voltage = 350 V and excitation slit = emission slit = 5 nm, the CD-H_2_O_2_-TMB-Apt-Urea system produced a scattering peak at 370 nm. Within 2.5–12.5 nmol/L, the RRS intensity had a linear relationship with urea concentration (Figures 4A,B).

### SEM and Laser Scattering of the System

**FIGURE 5:**
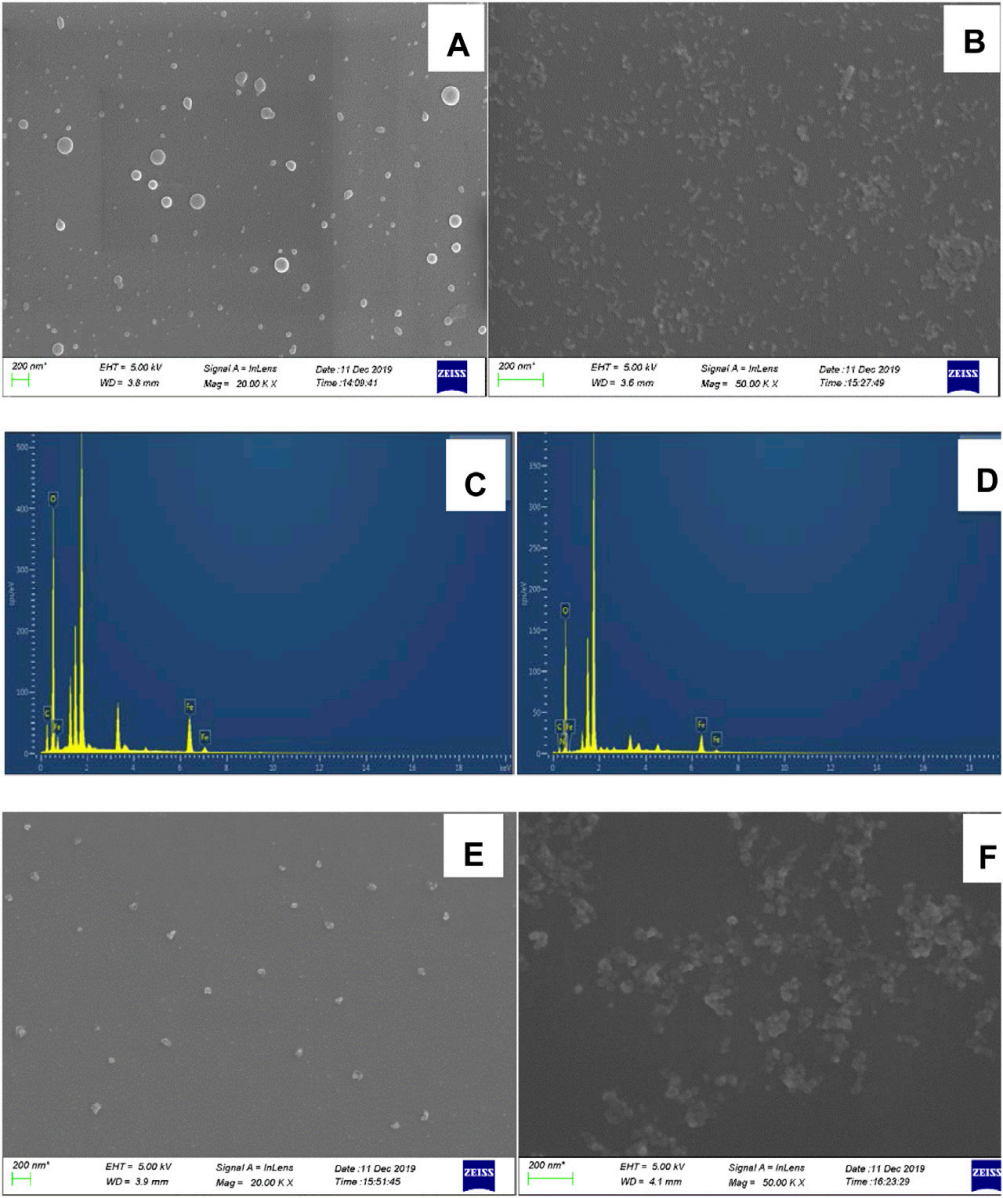
SEM and energy spectra images of analysis system. **(A)**: SEM of CD_Fe_; **(B)**: SEM of CD_FeN_; **(C)**: energy spectrum of CD_Fe_; **(D)**: energy spectrum of CD_FeN_; **(E)**: Analysis system without urea, 0.08 μg/ml CD_FeN_+ 5.0 μmol/L H_2_O_2_+ 0.03 mmol/L TMB+ 0.51 mmol/L pH 4.07 Tris-HCl+10 nmol/L Apt+0.08 mmol/L AgNPs; **(F)**: Analysis system with urea. E+12.5 nmol/L Urea.

SEM samples were prepared by dropping a small aliquot of each sample on the surface of a dried silicon wafer and allowing it to dry naturally. The average particle size of CD_Fe_ was approximately 50 nm (Figure 5A), and that of CD_FeN_ was approximately 30 nm (Figure 5B). CD_Fe_ and CD_FeN_ exhibited spectral peaks at 0.2, 5.2, and 5.5 keV corresponding to elemental Fe (Figure 5C, 5D); CD_FeN_ also showed a weak peak at 0.45 keV corresponding to N. Due to the presence of N, the CDs exhibited excellent catalytic activity. When no urea was added, the Apt_urea_ in the system wrapped the CD_FeN_, thereby inhibiting CD_FeN_ from catalyzing the oxidation of TMB by H_2_O_2_. As a result, fewer TMB_ox_ fluorescent probes were formed, and the extent of aggregation was low after adding AgNPs (aggregate size of 50 nm, Figure 5E). Upon addition of urea, CD_FeN_ encapsulation decreased, and the catalytic effect of the system was restored. As a result, the number of TMB_ox_ fluorescent probes formed gradually increased, and the extent of AgNPs-TMB_ox_ aggregation increased in the system to reach a size of 70 nm (Figure 5F), resulting in a linear increase in RRS intensity.

**FIGURE 6:**
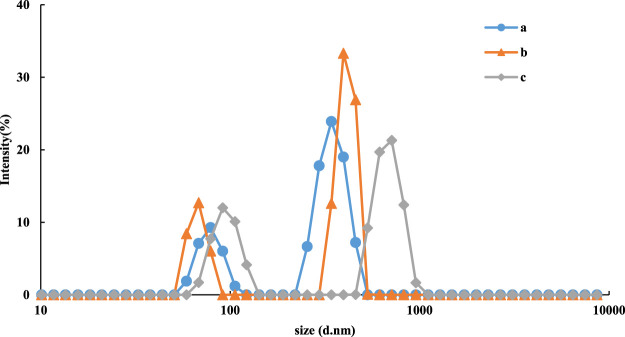
Particle size distribution of CD_FeN_-H_2_O_2_-TMB-Tris-HCl-Apt-Urea-AgNPs system. a: 0.08 μg/ml CDFeN+ 5.0 μmol/L H_2_O_2_+ 0.03 mmol/L TMB+ 0.51 mmol/L Tris-HCl (PH = 4.07)+10 nmol/L Apt+ Urea+0.08 mmol/LAgNPs; b: a+5.0 nmol/L Urea; c: a+12.5 nmol/L Urea.

Particle size analysis was used to determine the particle size distribution of the nanoparticles in the system. With increasing urea concentration, the target molecule urea and Apt_urea_ formed a stable conjugate and released the CDs, which restored their catalytic activity. Therefore, the product TMB_ox_ gradually increased along with TMB_ox_-AgNP aggregation. The particle sizes of the reaction product of the CD_FeN_-H_2_O_2_-TMB-Apt-Urea-AgNP system were 98, 110, and 130 nm, respectively (Figure 6), due to aggregation in the system.

### Optimization of Analysis Conditions

The effect of the experimental parameters on the SERS and FL signal intensities was systematically examined. As shown in Supplementary Figure S5 and in accordance with the experiments described in Section *Optimization and Characterization of CDs* containing 0.1 nmol/L urea, the use of a 0.51 mmol/L pH 4.07 Tris-HCl solution and 0.03 mmol/L TMB resulted in the strongest SERS signal. For the FL intensity, the optimized parameters were 5.0 μmol/L H_2_O_2_, 10 nmol/L Apt_urea_, 0.07 μg/ml CDs, 0.08 mmol/L AgNPs, a water bath temperature of 50°C, and a reaction time of 35 min.

### Working Curve

In this study, the FL, SERS, and RRS spectra of the CD-H_2_O_2_-TMB-Apt-Urea system were measured (Table 1). For the H_2_O_2_-TMB reaction, the catalytic effects of the two CDs (CD_Fe_ and CD_FeN_) were studied. The slope of the working curve corresponds to the catalytic ability of the CDs. The slope *K* of the working curve of the CD_FeN_-H_2_O_2_-TMB-Apt-Urea system was larger than that of the CD_Fe_ system, indicating that the former can be used for the FL detection of urea with a linear range of 3.33– 20 nmol/L and LOD of 1.0 nmol/L. Similar to FL, the SERS and RRS methods using the CD_FeN_ system were more sensitive than CD_Fe_ according to the evaluated slopes. Of the three modes, FL is simplest, as it does not require the addition of AgNPs, whereas SERS is the most sensitive. Compared with other reported urea analysis methods (Table 2) (Liu et al., 2010; Kumar et al., 2015; Chen et al., 2017a; Momenzadeh and Azadbakht, 2017; Mansouri and Azadbakht, 2019; Yarahmadi et al., 2019), this SERS method is more accurate and precise.

**TABLE 1:** The tri-mode analytical platform for assay of urea.

System	Methods	LR (nmol/L)	Regression equation	Coefficient	DL (nmol/L)
CD_Fe_-Urea	FL	3.33–20	ΔF_405 nm_ = 27.5C + 28.5	0.9856	1.4
	SERS	3.33–16.65	$\Delta I_{1598{\text{cm}}^{-1}}=166.3\text{C}+221.1$	0.9946	1.3
	RRS	2.5–12.5	ΔI_370 nm_ = 17.6C−2.9	0.9939	1.6
CD_FeN_-Urea	FL	3.33–20	ΔF_405 nm_ = 22.3C + 15.7	0.9880	1.0
	SERS	1.1–16.65	$\Delta I_{1598{\text{cm}}^{-1}}=90.8\text{C}+29.1$	0.9895	0.06
	RRS	2.5–12.5	ΔI_370 nm_ = 21.0C−6.5	0.9909	1.71

**TABLE 2:** Comparison of reported Urea analysis methods.

Method	Analysis principle	Linear range	Detection limit	Analysis characteristics	RSD (%)	Ref.
FL	The urea-specific DNA aptamer was isolated by an exponential enrichment method. In terms of inherent fluorescence differences and color changes, the aptamer sensor used unmodified gold nanoparticles (AuNP) to transduce the signal of aptamer-urea binding, thereby showed high selectivity to urea.	20–150 mM	20 mM	Simple operation and low sensitivity	—	36
Electrochemical	A mixture of carbon nanotubes and platinum nanoparticle-reduced graphene oxide (rGO) was used to surface modify the glassy carbon electrode (GCE). The urea aptamer was then immobilized on the nanocomposite by covalent bonding. Thus, aptamers with high affinity and selectivity for urea were used to quantify urea.	0.0–0.1 nM, 1.0–150 nM	1.9 pM	Complex operation and expensive equipment	7.9%	37
Electrochemical	Molecularly imprinted polymers (MIPs) also contained DNA aptamers on gold nanoparticles containing carbon nanotube networks (AuNP/CNT). The material was placed on a glass-carbon electrode (GCE), and GCE showed double recognition ability after removing urea from the MIP cavity. After the modified electrode was exposed to urea, the interface charge transfer of the redox probe hexacyanoferrate was measured under certain conditions. The change of the charge transfer resistance depended on the urea concentration, so the urea can be detected with high specificity.	0.005–0.1 nM, 1–500 nM	900 fM	Complex operation and high sensitivity	5.5%	38
Electrochemical	The primary amine was functionalized GO by a one-pot solvothermal method using ethylene glycol as the solvent and ammonia as the nitrogen precursor. Based on the signal amplification of carbon nanotubes/amine-functionalized GO as a sensing platform, and Apt as a probe, the label-free electrochemical analysis of urea was performed.	1–30 nM, 100–2000 nM	370 pM	Complex operation and high sensitivity	6.7–11.5%	39
SERS	The substrate made from Au/Cu hybrid nanostructure arrays was used to detect urea. Adjusting the gap size between adjacent nanorods to a sub-10 nm range produced high-density hot spots, which enables the substrate to detect urea signals at low concentrations.	—	1 mM	Simple operation and low sensitivity	9.5%	34
SERS-FL	Apt mediated the CD_FeN_ catalyzed H_2_O_2_ oxidation of TMB to form trifunctional probes of TMBox. The FL/SERS/Abs signals had a linear relationship with the concentration of Urea.	Flu:3.33–13.32 nmol/L; SERS:3.33–16.65 nnmol/L	1.12 nmol/L,1 nmol/L	Simple operation and high sensitivity	1.45–5.32%	This method

### Effects of Interfering Ions

The interference of coexisting ions on 10 nmol/L urea in the system was studied by RRS. The results indicated that 10 μmol/L K^+^, Cr^6+^, NH_4_^+^, Zn^2+^, SiO_3_^2−^, Mg^2+^, CO_3_^2−^, I^−^, Ca^2+^, and SO_4_^2−^; 5 μmol/L Al^3+^, Mn^2+^, Ba^2+^, Hg^2+^, Cu^2+^, and HSA; and 2 μmol/L Co^2+^, Fe^2+^, NO_2_^−^, Fe^3+^, Br^−^, Cr^3+^, and BSA did not interfere with the measurement. The interference of coexisting ions on 10 nmol/L urea in the system was then investigated by the SERS method. Similarly, the results showed that 10 μmol/L Ba^2+^, K^+^, Cr^6+^, NH_4_^+^, Zn^2+^, SiO_3_^2-^, Mg^2+^, Hg^2+^, CO_3_^2−^, I^−^, and Ca^2+^; 5 μg/L Al^3+^, Co^2+^, Mn^2+^, Cu^2+^, HSA, and SO_4_^2-^; and 2 μmol/L Fe^2+^, NO_2_^−^, Fe^3+^, Br^−^, Cr^3+^, and BSA did not interfere with the measurement. The interference of coexisting ions on 10 nmol/L urea in the system was studied by FL. The results indicated that 10 μmol/L K^+^, NH_4_^+^, Zn^2+^, SiO_3_^2−^, SO_4_^2−^, Mg^2+^, CO_3_^2−^, and Ca^2+^; 5 μmol/L Al^3+^, Mn^2+^, Fe^2+^, Ba^2+^, Hg^2+^, Cu^2+^ and NO_2_^−^; and 2 μmol/L Co^2+^, Br^−^, I^−^, Cr^3+^, HSA, and BSA did not interfere with the measurement. Therefore, our method has good selectivity.

### Real Sample Determination

Urea content determination plays an important role in the dairy industry, as its content in milk and dairy products should be less than 0.70 mg/g (Kumar et al., 2015). If the urea content in milk powder exceeds the normal range, consumption can lead to certain health problems. Thus, we detected the urea content in milk samples using our new method. Five milk samples were purchased from a supermarket and treated to obtain sample solutions according to an established method (Liang et al., 2019). The samples were then analyzed by the proposed method (the results are shown in Supplementary Table S2). The recovery rates were in the range of 96.4–106%, and the results of the proposed method for the determination of urea from milk samples were in agreement with the spectrophotometric method.

## Conclusion

In this study, a single reagent was used as a precursor to synthesize stable Fe/N-doped CDs (CD_FeN_) by a hydrothermal procedure. The molecular and spectral characteristics of the CD_FeN_ and their catalytic effect on the reaction of H_2_O_2_ and TMB were studied in detail using SERS, RRS, FL, and UV-vis absorption spectra. Apt_urea_ can adsorb onto the surface of the CD_FeN_ to turn off the nanocatalytic tri-mode indicator reaction. When the target molecule urea is added, Apt_urea_ releases the CD_FeN_ due to its specific binding, thereby restoring the CD_FeN_ catalytic activity to turn on the indicator reaction. With increasing urea concentration, the change in SERS/RRS/FL signals were linear, which establishes our method as a highly sensitive Apt-mediated, doped-CD, catalytic amplification, tri-spectroscopic platform.

## Data Availability

The original contributions presented in the study are included in the article/Supplementary Material, further inquiries can be directed to the corresponding authors.
